# Management of Adenocarcinoma in the Setting of Recently Operated Perianal Paget's Disease

**DOI:** 10.1155/2013/510813

**Published:** 2013-05-19

**Authors:** Margaret E. Clark, Andrew T. Schlussel, Ronald A. Gagliano

**Affiliations:** Department of General Surgery, Tripler Army Medical Center, 1 Jarrett White Road, Honolulu, HI 96859, USA

## Abstract

Perianal Paget's disease only rarely presents with a synchronous invasive anal or rectal cancer. The purpose of this study is to present a case of an otherwise healthy patient who developed perianal Paget's disease. He was then found to have an invasive rectal adenocarcinoma, after having undergone an extensive resection and reconstruction with a bilateral V-to-Y reconstruction. This report describes an overview of perianal Paget's disease, the management of this disease in association with anal or rectal cancer, and our patient's outcome.

## 1. Introduction

Extramammary Paget's disease (EMPD) is an uncommon condition, generally affecting Caucasian women with a peak incidence between the ages of 50 and 80. Despite a high recurrence rate, treatment is possible with wide local excision. Long-term survival in some reports is equivalent to the survival of the general population [1]. Prognosis is relatively favorable, with disease-free survival being 60–64% at 5 years [2, 3]. However, when there is an associated invasive anal or rectal cancer, prognosis is worse. 

Presenting signs and symptoms of perianal Paget's disease include eczema-like skin changes, itching, pain, the sensation of a lump, and bleeding. Pruritus ani is the most common symptom, occurring in 70% of patients. Paget's disease can be completely asymptomatic in 10% of patients [4, 5] and has even been incidentally diagnosed on hemorrhoidectomy specimens [1, 6]. In one study of eight cases, time from the onset of symptoms to diagnosis ranged between 6 and 96 months, with a median time of 27.3 months [2]. 

Histologically, perianal Paget's disease is an intraepithelial adenocarcinoma located within a 6 cm radius from the anal verge, below the dentate line. The mainstay of treatment is wide local excision, but other options include abdominoperineal resection, Mohs micrographic surgery, radiotherapy, chemoradiotherapy, or photodynamic therapy [2]. The rarity of perianal Paget's disease with an associated invasive component makes the management and surveillance of patients with this disease more difficult due to the lack of large, well-powered studies. The purpose of this report is to present a case of an otherwise healthy patient who developed perianal Paget's disease and subsequently was found to have invasive rectal adenocarcinoma after having undergone an extensive resection and reconstruction. 

## 2. Case Presentation

This is a case of a 44-year-old male who presented with severe pruritus ani, progressively painful bowel movements, hematochezia, and pain on rectal exam. He had a pertinent past medical history of hemorrhoidal disease that was diagnosed and confirmed on flexible sigmoidoscopy three years prior to presentation. On physical examination he had significant anal excoriation and therefore underwent a single punch biopsy of the anal margin. The biopsy was positive for extramammary Paget's disease, supported by positive staining with mucicarmine, periodic acid-Schiff (PAS), and polyclonal carcinoembryonic antigen (CEA). The biopsy was also negative for S100. 

**Figure 1:**
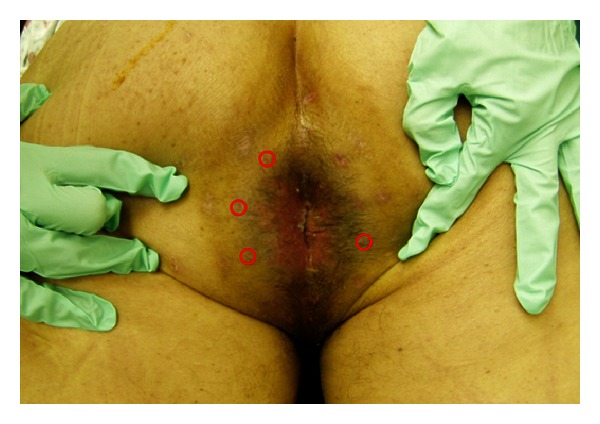
Mapping biopsies were taken circumferentially; four biopsies were positive for Paget's disease at the positions distinguished by red circles.

The patient subsequently underwent mapping biopsies from each quadrant at the dentate line, distal end of the anoderm, and one centimeter from the end of the anoderm. The biopsies confirmed extramammary Paget's disease without invasion. Due to the extensive positive results found on the first set of biopsies, a second procedure was performed. Twenty-four biopsies were obtained in a circumferential manner. The edge of the transition zone of the gross excoriation, as well as approximately four centimeters from the anal verge, was included for a radius of about one and a half centimeters (Figure 1). Four sites were positive for Paget's disease without invasive cancer. Further workup for metastatic disease to include colonoscopy, barium enema, and chest radiograph was negative. The patient was evaluated in a multidisciplinary tumor board and it was the consensus that surgical excision be attempted.

**Figure 2:**
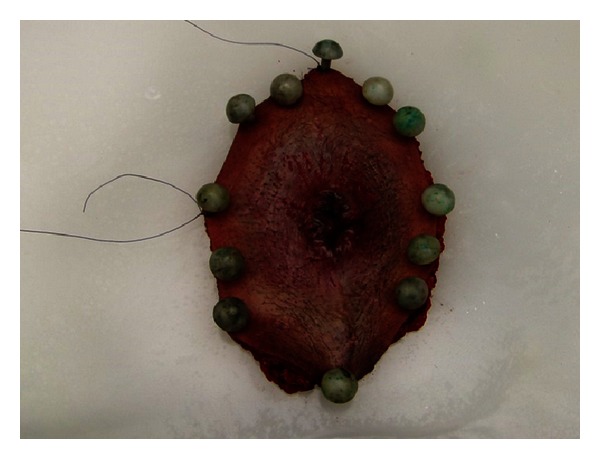
Wide local excision to include all positive biopsy sites was performed.

**Figure 3:**
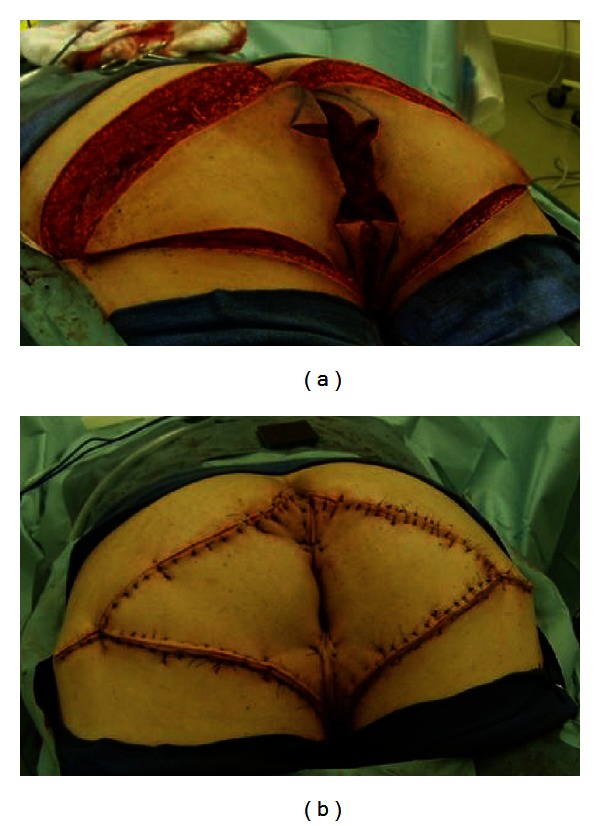
A V-to-Y gluteal fasciocutaneous flap reconstruction was performed.

**Figure 4:**
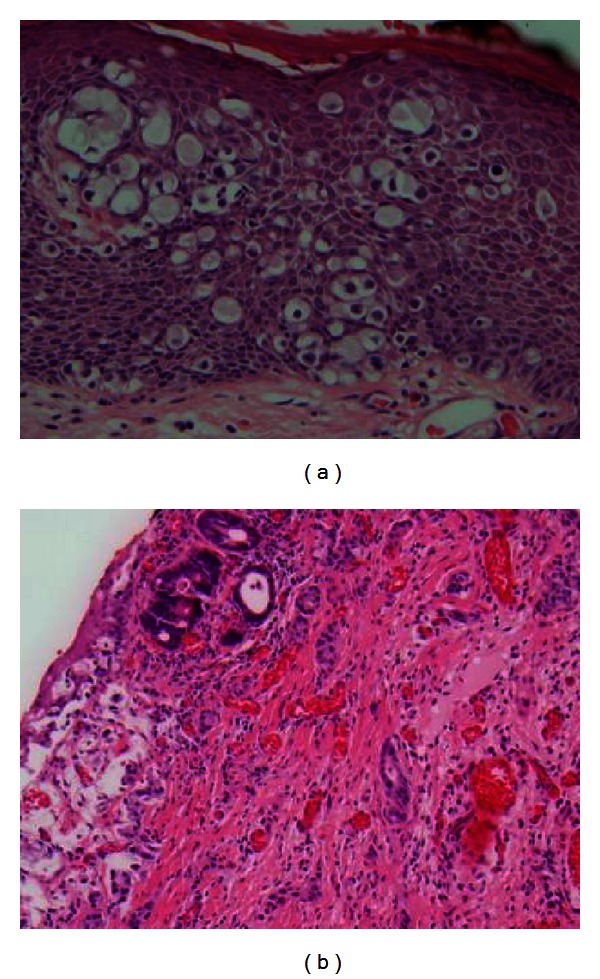
Extramammary Paget's disease associated with invasive adenocarcinoma of the anal canal, with focal mucinous features seen here.

A laparoscopy-assisted diverting loop ileostomy was performed first to protect the reconstruction. The patient was then placed in a jackknife position and a wide local excision to include the biopsy sites was performed (Figure 2). Dissection was taken down to the fascial level and the subcutaneous fat and skin were removed. The anus was dissected and excised just above the dentate line. A neo-anus was created using a bilateral V-to-Y gluteal fasciocutaneous flap closure (Figure 3). Histology was significant for extramammary Paget's disease associated with invasive adenocarcinoma of the anal canal with focal mucinous features (Figure 4). All skin margins were negative for Paget's disease. The proximal margin was focally positive for invasive adenocarcinoma at the left posterior lateral anorectal margin, with a final classification of a pT1N0 M0 stage I rectal adenocarcinoma.

With findings of advanced disease, the patient was recommended to have a repeat wide local excision, with or without radiation, and a mucosal advancement flap or abdominoperineal resection. The patient requested the most definitive operation with the lowest rate of recurrence. Therefore, an abdominoperineal resection with end colostomy was performed. Pathology was significant for no evidence of residual extramammary Paget's disease or invasive adenocarcinoma, and all lymph nodes were negative. 

**Figure 5:**
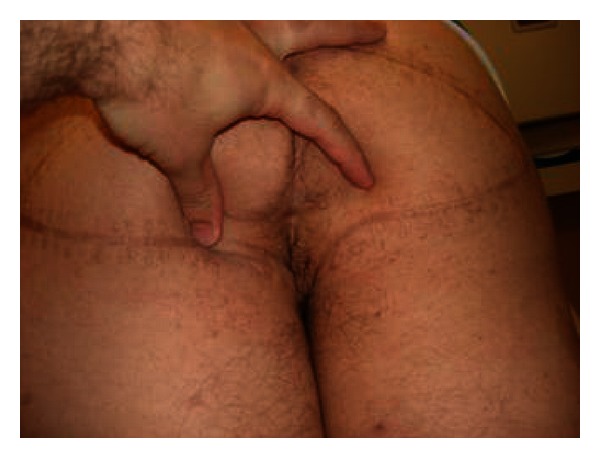
Final cosmetic outcome following abdominoperineal resection.

Postoperatively, his skin was surveyed every three months for the first two years then every six months (Figure 5). He had no subsequent excoriations; therefore no surveillance biopsies were obtained. The patient was last seen three and a half years after surgery only due to his relocation out of state. Imaging studies for recurrence and CEA levels remained negative. He is now disease-free six and half years following surgery.

## 3. Discussion

Perianal Paget's disease is a rare condition with only 193 cases reported between 1990 and 2008. Although this disease is typically a noninvasive intraepithelial lesion, treated with wide local excision, it has been associated with underlying malignancy, making the workup and staging of the disease critical prior to definitive treatment [7]. Underlying cancer can be found in up to 41.8% of patients with Paget's disease [2]. The disease has a tendency to relapse and may develop invasive features, metastasis or become associated with an anorectal adenocarcinoma. Recurrence rates have been reported to range from 33% to 86%, often due to incomplete excision [1, 7]. The disease-specific mortality is low if there is not an invasive component, but prognosis is poor when underlying adenocarcinoma is present, making the search for synchronous or metachronous tumors essential. 

Paget's disease arises mostly in areas rich in apocrine sweat glands. Lesions appear as well-demarcated red or brown plaques and often with scattered whitish or grayish lesions. There may be interspersed areas of clinically appearing normal skin, but these areas usually prove pathologically to be involved [8]. These lesions later become erosive, ulcerated, weeping, crusted, or scaly. Lesion size ranges from 1 to 90 cm^2^, with a median size of 12 cm^2^ [5]. Patients are often treated with an antifungal or a corticosteroid for a period of time before a biopsy is performed, delaying diagnosis. Lesions start close to the anus and then spread toward the perineum, genitalia, gluteal fold, buttocks, and, rarely, the anal canal [8]. 

The histogenesis is controversial, but there are four main theories [9]. The first is that Paget's disease cells may arise from underlying carcinoma of the apocrine or eccrine sweat glands by continuity. The second theory is that the cells are metastatic from an underlying rectal adenocarcinoma. The third suggests a simultaneous multicentric reaction in the epidermis, apocrine structures, and the glandular elements of the rectum from an unknown neoplastic, carcinogenic stimulus [2]. Lastly, Paget's disease may arise in situ from a faulty development of the pluripotential ectodermal basal cells. In one group of patient with underlying adenocarcinoma and a mucin-producing component, colonoscopy confirmed a disease-free distance between their underlying anorectal carcinoma and the Paget's disease, supporting metastasis as the origin. Mucin may also stimulate carcinogenesis of apocrine structures, resulting in infiltration of the epidermal barrier and formation of nests [2]. In our patient more than one biopsy was positive for mucin, and wide local excision showed focal mucinous features. This supports the theory of mucin playing a role in the pathogenesis of Paget's disease.

Signet ring cells are also present in perianal Paget's disease. These cells stain positive with periodic acid-Schiff mucin stain [2]. Primary EMPD cells predominantly show sweat gland differentiation CK20−/GCDFP15+. However, CK20+/GCDFP15− is also a possible presentation and tends to have a higher recurrence rate [10]. This pathology has been demonstrated in patients with associated rectal adenocarcinoma and endodermal differentiation with gastrointestinal-type glands [9]. The patient presented here was CK20+, which prompted us to be more aggressive in his treatment. EMPD can be associated with adenocarcinoma from retrograde skin invasion, or anorectal carcinoma with pagetoid diffusion in which the diseased cells are the cutaneous manifestation of adenocarcinoma. This case showed epidermal spread of invasive cancer in which the tumor cells have epidermotropic capacity and move from their origination to the skin [7]. 

Staining for S100, an acidic calcium-binding protein should not be positive in EMPD. Primary intraepidermal Paget's disease is usually CK7+, CK20−, and GCDFP-15+. With invasive disease the antigens are often seen in an opposite pattern: CK7−, CK20+, and GCDFP-15−. A decreased expression of MUC5AC and an overexpression of p53 and a fatty acid synthesis also correlate with advanced disease [8]. The majority of colon cancers reported in the literature are CK7−, but it is possible that CK7+ is more characteristic of rectal adenocarcinoma. The patient above was found to be CK7+ and CK20+.

In a series of eight cases of Paget's disease with underlying anorectal cancer, in all cases the underlying adenocarcinoma originated from the lower rectum and involved the dentate line [2]. Therefore, it is reasonable to take biopsies from the dentate line, which was done in this case. However, it was interesting there were no areas with invasion on biopsies prior to final surgical pathology in this case. 

The standard treatment for EMPD is wide local excision with deep extensions to remove the entire tumor bulk and all involved subcutaneous tissue. Wide local excision is difficult when the lesion extends into the anal canal. Temporary stomas are often indicated to allow for improved wound healing. Excision should extend at least 1 cm from the lesion and up to 2 cm if the margins are not clearly delineated. In the patient, the extent of dissection covered all positive biopsy sites, and skin margins were found to be negative of disease. Wide margins need to be obtained due to the potential for horizontal spread within the epidermis, beyond clinically apparent disease. These margins should be checked by frozen section before the end of the case [6]. If an underlying carcinoma is present, then abdominoperineal resection should be considered. Local application of 5-FU and bleomycin may reduce the margin of the lesions, but they are not sufficient alone for cure [8]. Systemic chemotherapy may be used if surgery and radiation are contraindicated. Radiation has been proposed for patients that are not good surgical candidates but has better success with Paget's disease without invasive cancer. Following wide local excision, functional results are acceptable and overall quality of life is similar to that of the general population [11]. A large proportion of patients report some form of fecal incontinence, but this is difficult to determine if it is a result of surgery or age related [11]. Another option for treatment is wide excisional biopsy and waiting for permanent sections to confirm the absence of invasive carcinoma before reconstruction. If the Paget's disease is known to be associated with an underlying carcinoma of the anus or rectum, the procedure of choice is an abdominoperineal resection with wide excision of the cutaneous lesion [9]. 

No consensus exists on the role of adjuvant therapy [1]. These patients need regular and close followup for longer than 5 years, as EMPD may metastasize via the lymphatic system. There are no set guidelines for followup, but proctosigmoidoscopy, punch biopsy of new lesions, and random biopsy at the edges of the resection at a yearly interval have been proposed [6]. Routine surveillance biopsies have also been discouraged unless the patient is symptomatic [3]. When patients develop a recurrence, repeat wide local excision plus radiotherapy if there is an invasive component is an option [12]. The patient described here elected to have an abdominoperineal resection to reduce the risk of needing future operations. This operation was successful even following a complex flap reconstruction. 

The high local recurrence rates are in part due to the irregular margins, multicentricity, and the tendency of the disease to involve what appears to be normal skin. Part of the wide local excision typically includes the anal canal, which requires a thinner flap for reconstruction. The mucosal defect, if large, needs to be reconstructed due to the risk of incontinence or stenosis of the anal tract due to scar formation [13]. A V-to-Y advancement flap or a posterior thigh trilobed flap may be used [13]. Because of the rarity of this disease, there is a lack of controlled clinical trials comparing treatments, so at this time surgery remains the mainstay of curative intent care. 

## 4. Conclusion

It is imperative to determine prior to surgery if perianal Paget's disease is associated with an underlying malignancy. The detection of a small foci of adenocarcinoma in the setting of perianal Paget's disease can be difficult. This patient had a complete evaluation to include colonoscopy and biopsies but was still found to have a small invasive component on final pathology. Delaying reconstruction until final pathologic evaluation after excision may allow for early definitive treatment and prevention of this scenario. However, staged APR is feasible after healing of primary excision and V-to-Y reconstruction, as demonstrated by this case. This patient has done well with no recurrence of disease six and a half years after surgery. 

## References

[1] Sarmiento JM, Wolff BG, Burgart LJ, Frizelle FA, Ilstrup DM (1997). Paget’s disease of the perianal region—an aggressive disease?. *Diseases of the Colon and Rectum*.

[2] Lian P, Gu WL, Zhang Z (2010). Retrospective analysis of perianal Paget’s disease with underlying anorectal carcinoma. *World Journal of Gastroenterology*.

[3] McCarter MD, Quan SHQ, Busam K, Paty PP, Wong D, Guillem JG (2003). Long-term outcome of perianal Paget’s disease. *Diseases of the Colon and Rectum*.

[4] Shepherd V, Davidson EJ, Davies-Humphreys J (2005). Extramammary Paget’s disease. *BJOG*.

[5] Zollo JD, Zeitouni NC (2000). The Roswell Park Cancer Institute experience with extramammary Paget’s disease. *British Journal of Dermatology*.

[6] Beck DE, Fazio VW (1987). Perianal Paget’s disease. *Diseases of the Colon and Rectum*.

[7] Minicozzi A, Borzellino G, Momo R, Steccanella F, Pitoni F, De Manzoni G (2010). Perianal Paget’s disease: presentation of six cases and literature review. *International Journal of Colorectal Disease*.

[8] Kanitakis J (2007). Mammary and extramammary Paget’s disease. *Journal of the European Academy of Dermatology and Venereology*.

[9] Gaertner WB, Hagerman GF, Goldberg SM, Finne CO (2008). Perianal Paget’s disease treated with wide excision and gluteal skin flap reconstruction: report of a case and review of the literature. *Diseases of the Colon and Rectum*.

[10] Goldblum JR, Hart WR (1998). Perianal Paget’s disease: a histologic and immunohistochemical study of 11 cases with and without associated rectal adenocarcinoma. *American Journal of Surgical Pathology*.

[11] Conklin A, Hassan I, Chua HK (2009). Long-term functional and quality of life outcomes of patients after repair of large perianal skin defects for paget’s and bowen’s disease. *Journal of Gastrointestinal Surgery*.

[12] Marchesa P, Fazio VW, Oliart S, Goldblum JR, Lavery IC, Milsom JW (1997). Long-term outcome of patients with perianal Paget’s disease. *Annals of Surgical Oncology*.

[13] Kishi K, Nakajima H, Imanishi N, Nakajima T (2010). Anal and perianal reconstruction after extramammary Paget’s disease using a posterior thigh flap with a thin square wing. *Journal of Plastic, Reconstructive and Aesthetic Surgery*.

